# Comparison Bioinformatic Analysis of Extracellular Vesicles-Related Genes and MicroRNAs in Breast Cancer

**DOI:** 10.3390/ijms26125906

**Published:** 2025-06-19

**Authors:** Durmus Ayan, Serife Buket Bozkurt Polat, Esma Ozmen, Mehmet Ali Gul

**Affiliations:** 1 Faculty of Medicine, Medical Biochemistry, Nigde Omer Halisdemir University, 51100 Nigde, Türkiye; buketbozkurt@yahoo.com (S.B.B.P.);; 2 Faculty of Medicine, Medical Biochemistry, Amasya University, 05200 Amasya, Türkiye; mehmetali.gul@amasya.edu.tr

**Keywords:** extracellular vesicles, breast cancer, biomarkers

## Abstract

Breast cancer (BC) remains a leading cause of cancer-related mortality in women, with treatment challenges due to the lack of targeted therapies. Extracellular vesicles (EVs) play a crucial role in BC progression by carrying bioactive molecules. This study analyzed EV-associated molecules (ENPEP, TIMP1, CD36, MARCKS, DAB2, CXCL14, miR-181b-5p, miR-222-3p) using bioinformatics tools. We used GEPIA2; Human Protein Atlas (HPA) 24.0; bc-GenExMiner v5.1; UALCAN 2022; Kaplan–Meier plotter 2025; ENCORI database v2.0; Enrichr-KG web tool 2021; Cancer Hallmark Enrichment tool 2025; Tumor, Normal, and Metastatic (TNM) plot database 2025; MicroRNA Target Prediction Database 6.0; TargetScan 8.0; and STRING database 12.0. CD36, DAB2, and CXCL14 were significantly downregulated, while TIMP1 was upregulated in BC tissues (*p* < 0.05). CD36, CXCL14, and DAB2 were predominantly low in triple-negative and basal-like subtypes, whereas TIMP1 was higher in HER2+, ER+, and PR+ tumors (*p* < 0.01). These changes correlated with promoter methylation patterns. Higher TIMP1, DAB2, and CXCL14 levels were associated with improved overall survival (*p* < 0.05). miR-222-3p was downregulated and positively correlated with TIMP1 and DAB2, while miR-181b-5p was upregulated and negatively correlated with CXCL14. TNM analysis confirmed these expression changes. Functional enrichment linked these molecules to key cancer hallmarks, including proliferation and angiogenesis. CD36, DAB2, CXCL14, TIMP1, miR-222-3p, and miR-181b-5p may serve as biomarkers for BC pathogenesis and potential therapeutic targets. Further studies are needed to validate these findings.

## 1. Introduction

Breast cancer (BC) is one of the most common malignancies among women worldwide and remains a leading cause of cancer-related death, despite advances in detection and treatment strategies [1]. The heterogeneity of BC and the development of resistance to therapy continue to pose major clinical challenges [2,3]. Therefore, identifying new molecular markers and understanding the mechanisms that drive disease progression are crucial for improving patient outcomes.

Extracellular vesicles (EVs), especially exosomes, have emerged as key players in cancer biology. These lipid-bound vesicles are secreted by cells and serve as carriers of bioactive molecules, including proteins, lipids, microRNAs (miRNAs), and long non-coding RNAs (lncRNAs) [4]. EVs play a pivotal role in intercellular communication, influencing tumor proliferation, angiogenesis, immune modulation, and metastasis [5].

Recent evidence indicates that EVs reflect the molecular characteristics of their cell of origin. In BC, the cargo of EVs—particularly regulatory RNAs and proteins—has been implicated in promoting tumor aggressiveness and therapy resistance [6]. For example, exosomal miRNAs can activate pro-metastatic signaling pathways or remodel the tumor microenvironment. Thus, EV content profiling may represent a promising source of non-invasive biomarkers for early detection, prognosis, and therapeutic monitoring [7].

Although several studies have examined the role of EVs in BC, most have focused on limited sets of molecules or lacked comprehensive bioinformatic integration. The molecular mechanisms regulating EV cargo selection and secretion also remain insufficiently understood. These gaps limit our ability to fully exploit EVs as diagnostic or therapeutic tools [8,9,10,11].

EVs, including exosomes and microvesicles, are lipid bilayer-enclosed particles secreted by virtually all cell types and play critical roles in intercellular communication. They facilitate the horizontal transfer of bioactive molecules such as proteins, mRNAs, and lncRNAs that can modulate the tumor microenvironment and influence cancer progression [12]. Several molecules identified in our study, including TIMP1, CD36, DAB2, and miR-181b-5p, have been previously reported to be enriched in EVs derived from BC cells, as documented in EV-specific repositories such as Vesiclepedia and ExoCarta [13,14]. These EV-associated molecules are known to contribute to hallmark cancer processes such as proliferation, angiogenesis, metastasis, and immune modulation [15]. Therefore, we focused our analysis on these EV-linked targets to explore their expression patterns, prognostic relevance, and potential functional interactions in BC subtypes.

In recent years, bioinformatics-based approaches have emerged as indispensable tools for unraveling the complex molecular networks underlying cancer progression. These analyses in silico facilitate the integration and interpretation of large-scale omics data from various public repositories, such as TCGA and GEO. By leveraging such datasets, researchers can systematically identify differentially expressed genes, non-coding RNAs, and signaling pathways that may serve as potential biomarkers or therapeutic targets. Importantly, these computational frameworks provide a cost-effective and scalable alternative to wet-lab experiments, allowing for hypothesis generation and validation without the immediate need for direct experimental assays [16]. This study is entirely based on bioinformatics analyses, with no experimental procedures conducted. The authors relied exclusively on previously published, publicly available datasets to investigate EV-associated molecules in BC. In this study, we aimed to address this gap by systematically evaluating a panel of EV-associated molecules in BC using integrated bioinformatics tools. We focused on ENPEP, TIMP1, CD36, MARCKS, DAB2, CXCL14, miR-181b-5p, and miR-222-3p—genes and miRNAs previously implicated in cancer but not comprehensively analyzed in the context of EVs in BC. Our approach contrasts with prior work by combining multiple datasets and tools to assess gene expression, prognostic significance, and potential regulatory interactions.

In order to investigate the expression and regulation of EV-related genes in BC, we systematically selected a panel of molecules (ENPEP, TIMP1, CD36, MARCKS, DAB2, CXCL14, miR-181b-5p, and miR-222-3p) based on their documented significance in the literature and their relevance to EV biology and BC pathogenesis. We utilized comprehensive public databases, including GEPIA2; Human Protein Atlas (HPA) 24.0; bc-GenExMiner v5.1; UALCAN 2022; Kaplan–Meier plotter 2025; ENCORI database v2.0; Enrichr-KG web tool 2021; Cancer Hallmark Enrichment tool 2025; Tumor, Normal, and Metastatic (TNM) plot database 2025; MicroRNA Target Prediction Database 6.0; TargetScan 8.0; and STRING database 12.0. These resources facilitated the assessment of gene/microRNA expression patterns, methylation status, prognostic significance, and molecular interactions across various BC subtypes.

## 2. Results

To explore the relevance of EV-associated molecules in BC, we performed a comprehensive multi-omics analysis integrating transcriptomic expression, subtype-specific profiling, promoter methylation, prognostic impact, and functional enrichment. We began by assessing the differential expression of selected EV-associated genes and miRNAs between tumor and normal breast tissues. This was followed by correlation analysis to investigate potential regulatory interactions. We then examined their prognostic significance using survival analysis, explored epigenetic regulation via promoter methylation status, and assessed functional implications through enrichment of biological pathways and cancer hallmarks. This sequential approach enabled us to characterize the expression behavior, clinical relevance, and mechanistic roles of these candidate molecules in the BC context.

### 2.1. Expression Profile Results

**Figure 1:**
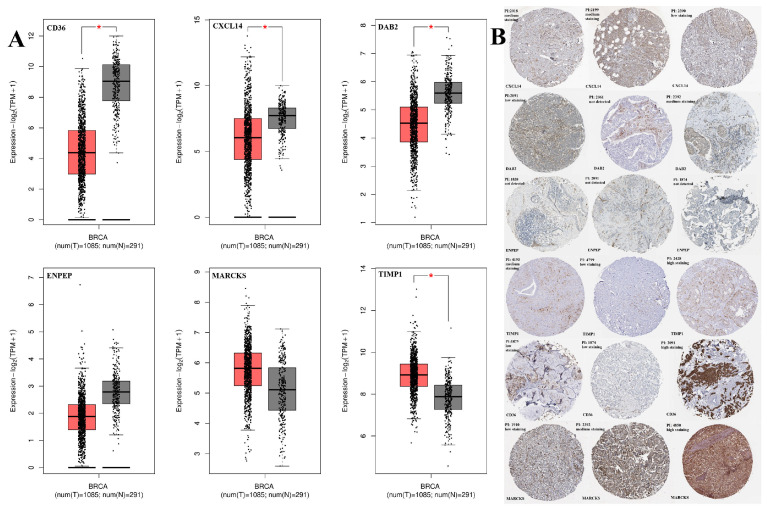
(**A**) Boxplot of the ENPEP, TIMP1, CD36, MARCKS, DAB2, and CXCL14 gene expression results in breast cancer were demonstrated via GEPIA2 webtool. * *p* < 0.05 is statistically significant. Each point represents an individual sample (n = 1085 tumor samples, 291 normal tissues) based on RNA-seq data from the TCGA-BRCA and GTEx datasets accessed via the GEPIA2 platform. BC tissue (red) and normal breast tissues (gray). (**B**) Validation of the genes using the Human Protein Atlas (HPA) database.

CD36, DAB2, and CXCL14 were statistically downregulated while TIMP1 was upregulated in the BC cohort compared to normal adjacent tissue (*p* < 0.05). However, there was no statistical change in ENPEP and MARCKS (*p* > 0.05) (Figure 1A). Additionally, Immunohistochemistry (IHC) evaluation results of the genes examined in HPA are shown in Figure 1B. These findings highlight significant alterations in EV-associated genes, whose differential expression in BC may reflect functional implications in EV-mediated communication.

### 2.2. Gene Correlation and Targeted Expression Analysis Results

**Figure 2:**
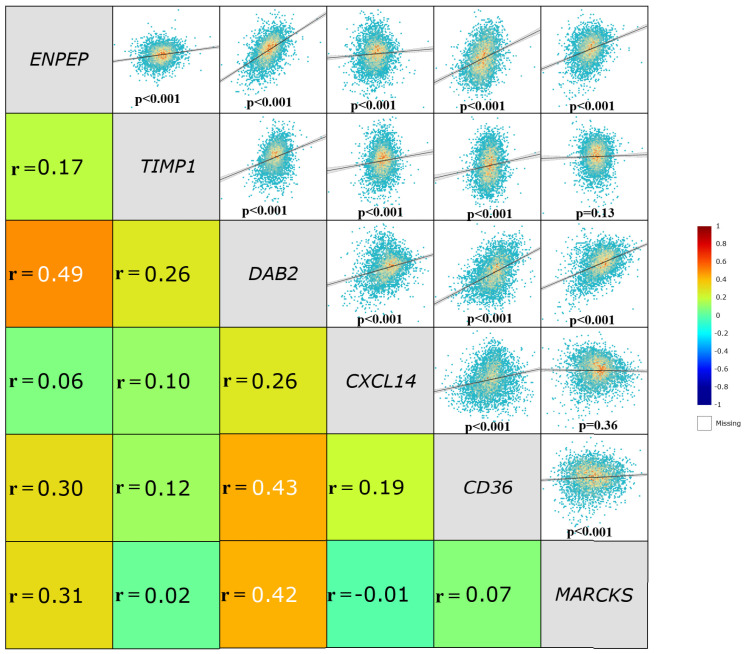
The correlations of the examined genes with each other. Spearman correlation of the examined genes with each other in breast cancer tissues (n = 1085). Data were retrieved from TCGA-BRCA and analyzed using the ENCORI database.

Additionally, the correlations of the examined genes with each other are shown in Figure 2. Accordingly, while no correlation was found between TIMP1 and MARCKS or between CXCL14 and MARCKS (*p* = 0.13 and *p* = 0.36, respectively), a positive significant correlation was observed between all the others (*p* < 0.001).

**Figure 3:**
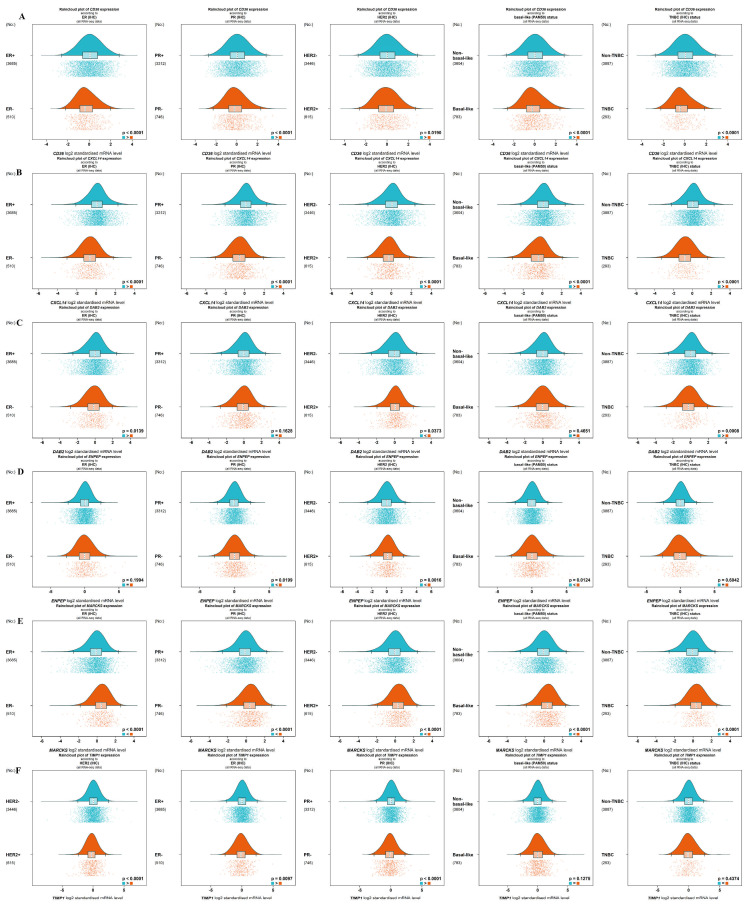
All RNA-seq data results according to receptor status (ER, PR, HER2) and intrinsic molecular subtypes (TNBC and basal-like): (**A**) CD36, (**B**) CXCL14, (**C**) DAB2, (**D**) ENPEP, (**E**) MARCKS, (**F**) TIMP1.

Five criteria are suggested for categorizing tumors: estrogen receptor (ER) via IHC, progesterone receptor (PR) via IHC (all **RNA-seq data**), HER2 status via IHC (all **RNA-seq data**), intrinsic molecular subtypes determined by PAM50 (all **RNA-seq data**), triple-negative breast cancer (TNBC) identified through IHC (all **RNA-seq data**), and basal-like subtypes identified through PAM50. The analysis revealed that the genes CD36, CXCL14, DAB2, MARCKS, and ENPEP exhibited low expression levels, particularly in TNBC and basal-like subtypes (*p* < 0.01). Conversely, the TIMP1 gene demonstrated high expression in HER2+, ER+, and PR+ tumors (*p* < 0.01). Specifically, the *CD36* gene showed significantly reduced expression in ER-, PR-, HER2+, TNBC, and basal-like groups (*p* < 0.001, *p* < 0.001, *p* = 0.0190, *p* < 0.001, *p* < 0.001, respectively) as illustrated in Figure 3A. The CXCL14 gene exhibited low expression levels in ER-, PR-, HER2+, TNBC, and basal-like tumors (*p* < 0.001) (Figure 3B). The DAB2 gene exhibited low expression in ER- and TNBC tumors (*p* = 0.0139, *p* = 0.0008, respectively), while it showed high expression levels in HER2+ tumors (*p* = 0.0373) (Figure 3C). The ENPEP gene was detected at significantly higher levels in PR-, HER2+, and basal-like tumors (*p* = 0.0199, *p* = 0.0016, *p* = 0.0124, respectively) (Figure 3D). The MARCKS gene demonstrated high expression in ER-, PR-, HER2+, TNBC, and basal-like tumors (*p* < 0.001) (Figure 3E). The TIMP1 gene exhibited low levels of expression in HER2+, ER-, and PR- tumors (*p* < 0.001, *p* = 0.0097, *p* < 0.001, respectively) (Figure 3F).

**Figure 4:**
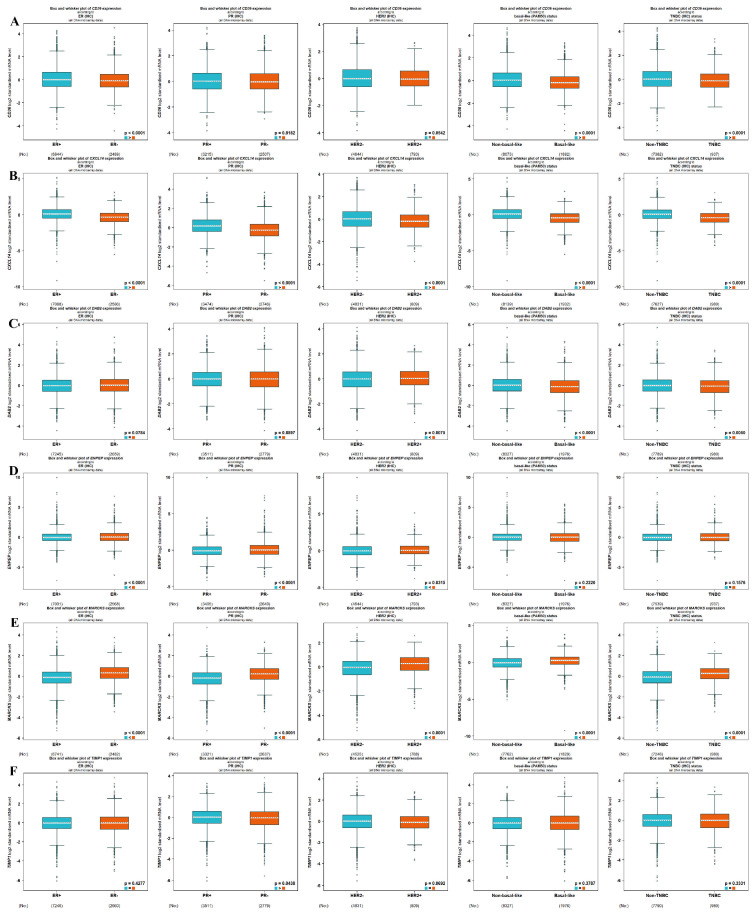
All **DNA microarrays** data results according to receptor status (ER, PR, HER2) and intrinsic molecular subtypes (TNBC and basal-like): (**A**) CD36, (**B**) CXCL14, (**C**) DAB2, (**D**) ENPEP, (**E**) MARCKS, (**F**) TIMP1.

Five criteria are suggested for categorizing tumors: ER via IHC, PR via IHC (all **DNA microarrays)**, HER2 status via IHC (all **DNA microarrays)**, intrinsic molecular subtypes determined by PAM50 (all **DNA microarrays)**, TNBC identified through IHC (all **DNA microarrays)**, and basal-like subtypes identified through PAM50. Specifically, the CD36 gene showed significantly reduced expression in ER-, TNBC, and basal-like groups (*p* < 0.001, *p* < 0.001, *p* < 0.001, respectively) as illustrated in Figure 4A. The CXCL14 gene showed decreased expression levels in ER-, PR-, HER2+, TNBC, and basal-like tumors (*p* < 0.001) (Figure 4B). The DAB2 gene exhibited low expression in ER-, PR-, HER2+, TNBC, and basal-like tumors (*p* < 0.001) (Figure 4C). The ENPEP gene was detected at significantly lower levels in HER2, TNBC, and basal-like tumors (*p* = 0.0075, *p* < 0.001, *p* < 0.001, respectively) (Figure 4D). The MARCKS gene demonstrated high expression in ER-, PR-, HER2+, TNBC, and basal-like tumors (*p* < 0.001) (Figure 4E). The TIMP1 gene exhibited low levels of expression in PR- tumors (*p* = 0.0438, respectively) (Figure 4F).

### 2.3. The Promotor Methylation Analysis

**Figure 5:**
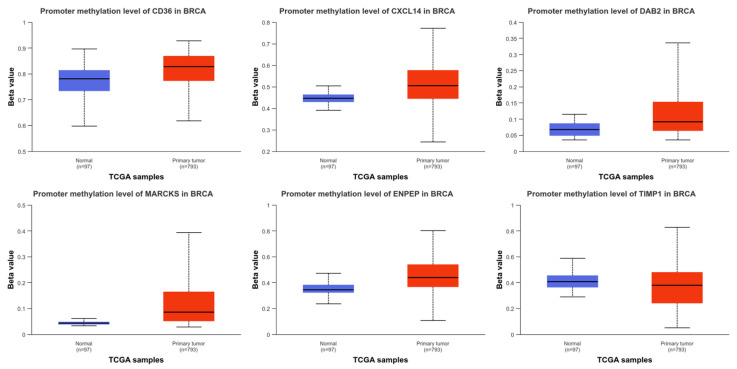
Boxplots showing the promoter methylation levels (beta values) of **CD36**, **CXCL14**, **DAB2**, **MARCKS**, **ENPEP**, and **TIMP1** in BRCA samples. Methylation data were obtained from The Cancer Genome Atlas (TCGA) using the UALCAN platform. Comparisons were made between normal breast tissues (n = 97) and primary tumor tissues (n = 793). Beta values range from 0 (unmethylated) to 1 (fully methylated).

**Figure 6:**
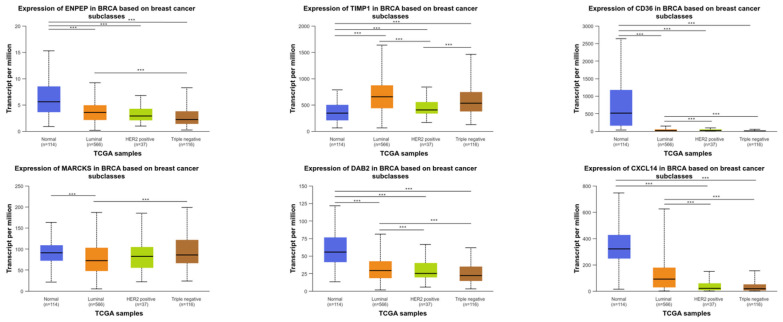
Boxplots showing the transcript per million (TPM) expression levels of **ENPEP**, **TIMP1**, **CD36**, **MARCKS**, **DAB2**, and **CXCL14** across different breast cancer molecular subtypes: **luminal**, **HER2-positive**, **triple-negative**, and **normal tissues**. Expression data were obtained from The Cancer Genome Atlas (TCGA) using the UALCAN platform. The number of samples in each group is indicated: normal (n = 114), luminal (n = 611), HER2-positive (n = 82), and triple-negative (n = 120). Asterisks denote statistically significant differences (*p* < 0.05) between the indicated groups.

CD36, CXCL14, and DAB2 were statistically hypermethylated, while TIMP1 was hypomethylated in BC compared to normal (*p* < 0.05). However, there was no statistical change in ENPEP or MARCKS (*p* > 0.05) (Figure 5). As a result of the examination conducted in BC subgroups, downregulation in expression profiles generally occurred, while upregulation in TIMP1 expression levels was detected compared to normal adjacent tissue (Figure 6).

### 2.4. The Overall Survival Status

**Figure 7:**
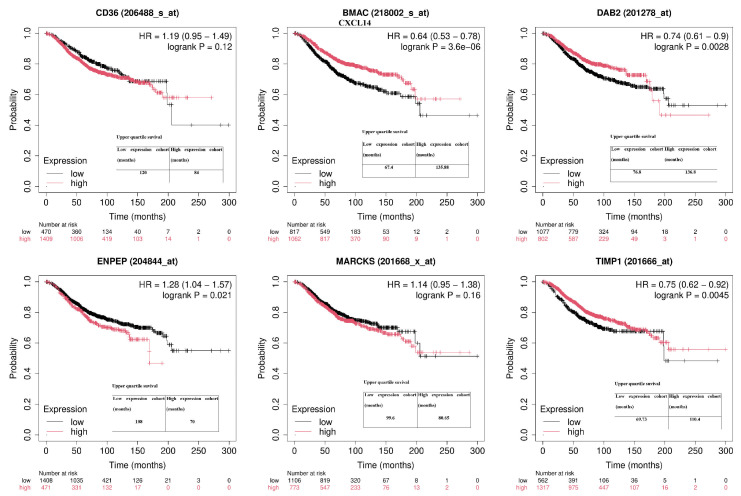
Kaplan–Meier overall survival analysis for six extracellular vesicle (EV)-associated genes: **CD36**, **CXCL14**, **DAB2**, **ENPEP**, **MARCKS**, and **TIMP1**. Patients were divided into high- and low-expression groups based on the median expression threshold. Survival curves were generated using the KM-Plotter 2025 tool utilizing Affymetrix probe data for BRCA patients (n ≈ 1900 total). Hazard ratios (HRs), 95% confidence intervals (CIs), and log-rank *p*-values are shown for each gene.

**Figure 8:**
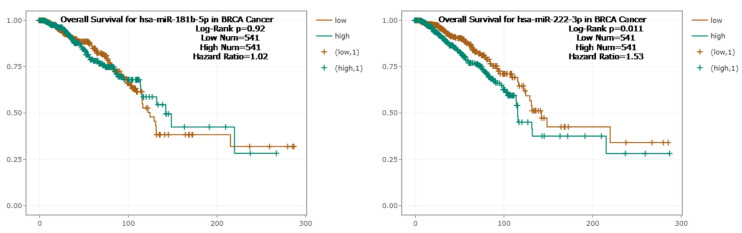
Kaplan–Meier overall survival analysis for hsa.miR-222-3p expression in breast cancer patients. A total of 1082 samples were analyzed using the KM-Plotter database, with patients divided into high- and low-expression groups based on the median cutoff.

The elevated expression levels of TIMP1, DAB2, and CXCL14 were statistically associated with a longer OS (*p* < 0.05). However, no significant association was observed between the expression of ENPEP, CD36, and MARCKS and overall survival (*p* > 0.05) (Figure 7). A decreased expression level of miR-222-3p was associated with a longer OS (*p* = 0.011). However, no significant association was observed between miR-181b-5p expression and overall survival (*p* > 0.05) (Figure 8).

### 2.5. The Relationship Between MicroRNA vs. BC and MicroRNA vs. RNA

**Figure 9:**
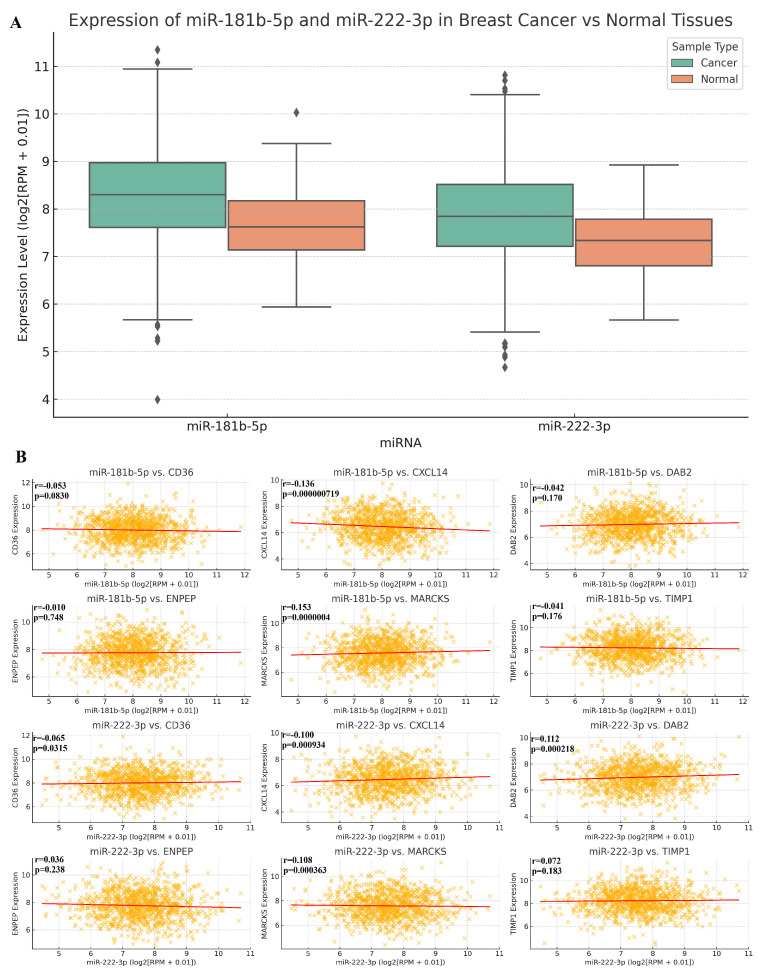
(**A**) Boxplots showing the differential expression levels of hsa-miR-181b-5p and hsa-miR-222-3p in breast cancer tissues (n = 1085) compared to normal breast tissues (n = 104) based on ENCORI project data. Expression is presented in log_2_(RPM + 0.01). (**B**) Correlation analysis between hsa-miR-181b-5p or hsa-miR-222-3p and selected EV-associated target genes (CD36, CXCL14, DAB2, ENPEP, MARCKS, TIMP1) in BRCA samples (n = 1085). Pearson correlation coefficients (r) and *p*-values are shown. Data were obtained from the ENCORI database. Each dot represents an individual sample.

miR-222-3p was statistically downregulated while miR181b-5p was upregulated in the BC cohort compared to normal adjacent tissue (*p* < 0.05). miR181b-5p was negatively correlated with CXCL14 while miR-222-3p was positively correlated with TIMP1, CD36, and DAB2 (*p* < 0.05). miR181b-5p was upregulated and showed a statistically significant negative correlation with CXCL14 (*p* = 0.00000719), suggesting an inverse expression pattern, while it was positively correlated with MARCKS (*p* = 0.00000040), indicating a possible co-expression. miR-222-3p was positively correlated with TIMP1, MARCKS, and TIMP1 (*p* = 0.000208, *p* = 0.000363, *p* = 0.0183, respectively), while it was negatively correlated with CD36 and CXCL14 (*p* = 0.0315, *p* = 0.000934, respectively) (Figure 9).

### 2.6. Gene Set Enrichment Analysis of ENPEP, TIMP1, CD36, MARCKS, DAB2, and CXCL14

**Figure 10:**
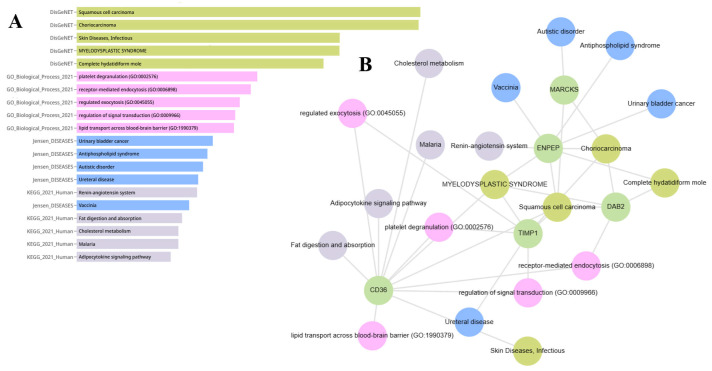
(**A**) Bar chart representing the top enriched biological processes, molecular functions, and signaling pathways associated with the selected EV-related genes (ENPEP, TIMP1, CD36, MARCKS, DAB2, CXCL14), as identified through functional annotation using the Enrichr-KG and Cancer Hallmarks Enrichment platforms. Categories include GO terms, KEGG pathways, and hallmark gene sets. (**B**) Network visualization showing the association of EV-related genes with enriched disease phenotypes and biological functions based on integrated enrichment analysis. Nodes represent genes (green), pathways or biological processes (pink/lavender), and disease terms (blue), with edges indicating reported associations from curated databases. This network suggests shared functional roles and pathological relevance of these EV cargo molecules in cancer-related and immune-modulatory processes.

According to KEGG pathway analyses, CD36 is a component of the cholesterol metabolism pathway, ENPEP is part of the renin-angiotensin system, and DAB2 is involved in the fat digestion and absorption pathway. Data from the Jensen Lab indicate that the MARCKS gene is associated with autistic disorder. ENPEP is linked to vaccinia, antiphospholipid syndrome, and bladder cancer. TIMP1 is related to ureteral diseases. DisGeNET data reveal associations between myelodysplastic syndrome and the genes CD36, DAB2, ENPEP, and TIMP1. Complete hydatidiform mole is associated with the DAB2 and ENPEP genes. Squamous cell carcinoma is linked to the expression of MARCKS, CD36, DAB2, ENPEP, and TIMP1. Choriocarcinoma is associated with MARCKS, DAB2, ENPEP, and TIMP1. Infectious skin diseases are associated with CD36 expression. Enrichment analysis results of ENPEP, TIMP1, CD36, MARCKS, DAB2, and CXCL14 are shown in Figure 10A,B. The enrichment analysis of these EV-linked genes suggests that their altered expression patterns may influence cancer-related pathways via extracellular vesicle transport mechanisms.

### 2.7. Tumor, Normal, and Metastatic (TNM) Analysis

**Figure 11:**
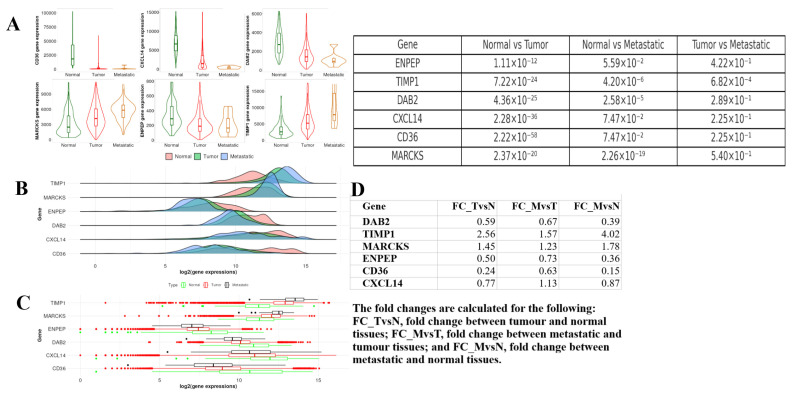
(**A**) Violin plots displaying the expression levels of the selected genes (ENPEP, TIMP1, DAB2, CXCL14, CD36, MARCKS) across normal, tumor, and metastatic samples. Dunn’s test was performed to assess significant differences between groups, with *p*-values indicated above each plot. (**B**) Density plots showing the distribution of gene expression values for each gene across the three sample types (normal, tumor, metastatic). (**C**) Boxplots illustrate the gene expression values across normal, tumor, and metastatic groups. Outliers are indicated by red and green dots, representing significant differences in expression between sample groups. (**D**) Multivariate Cox regression analysis results displaying the fold changes in gene expression for DAB2, TIMP1, MARCKS, ENPEP, CD36, and CXCL14 across normal versus tumor tissues (FC_TvsN), normal versus metastatic tissues (FC_MvsN), and tumor versus metastatic tissues (FC_MvsT).

The expression levels of the genes CD36, CXCL14, DAB2, MARCKS, ENPEP, and TIMP1 in normal, tumor, and metastatic tissues were analyzed using various statistical methods. Violin and box plots were employed to illustrate the expression levels of each gene across these tissue types. The Dunn test results indicated significant expression changes between normal and tumor tissues for all genes (*p* < 0.05). Notably, significant differences were observed for the CD36 (*p* = 2.22 × 10^−58^) and DAB2 (*p* = 4.36 × 10^−25^) genes. The differences between tumor and metastatic tissues were gene-specific; for instance, no significant changes were observed in some genes, such as CD36 and ENPEP (*p* = 0.42), whereas significant changes were detected in others, such as MARCKS (*p* = 5.40 × 10^−7^). A multivariate Cox regression analysis was conducted to assess the potential roles of these genes in cancer progression. Fold change (FC) values were used to reflect gene expression changes during the transition from normal tissue to tumor and metastatic stages. The DAB2 gene exhibited a 0.59-fold decrease between tumor and normal tissues, with a measurement of 0.67 in metastatic tissues. TIMP1 showed a 2.56-fold increase in tumors and a 1.57-fold increase in the metastatic stage, resulting in a total change of 4.02-fold. CD36 demonstrated a significant decrease (0.24) during the transition from normal to tumor, with an even more pronounced decrease in the metastatic stage (FC_MvsN = 0.15) (Figure 11).

### 2.8. Cancer Hallmark Enrichment Analysis

**Figure 12:**
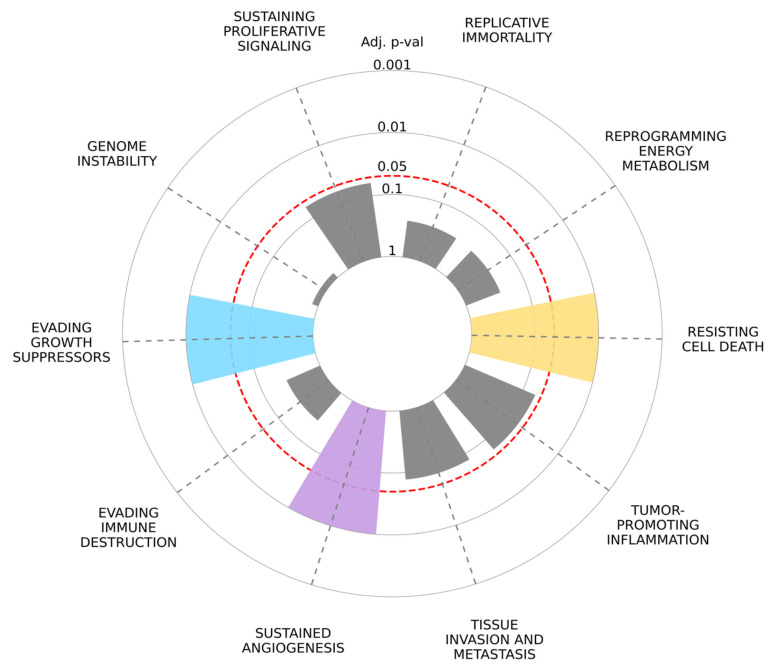
Radial bar plot illustrating the enrichment significance of cancer hallmark pathways associated with the selected EV-related genes. Each bar represents an individual hallmark process, with bar height indicating the adjusted *p*-value (log scale) derived from enrichment analysis. Hallmarks such as **evading growth suppressors**, **evading immune destruction**, **resisting cell death**, and **sustained angiogenesis** were significantly enriched (adjusted *p* < 0.05, highlighted in color). The red dashed line denotes the 0.05 significance threshold. Data were obtained using the Cancer Hallmark Enrichment 2025 tool based on curated gene sets linked to breast cancer pathogenesis.

Genes such as TIMP1, ENPEP, CD36, and CXCL14 have been identified as contributors to the mechanism of “sustaining proliferative signaling” (adj. *p* = 0.06175). Additionally, the genes CXCL14, DAB2, ENPEP, and MARCKS are significant in the “evading growth suppressors” category (adj. *p* = 0.01012). Furthermore, the genes TIMP1, CD36, and CXCL14 are implicated in the “sustained angiogenesis” mechanism (adj. *p* = 0.01012) (Figure 12 and Table 1).

### 2.9. Result of miRNAs Associated with Genes

**Figure 13:**
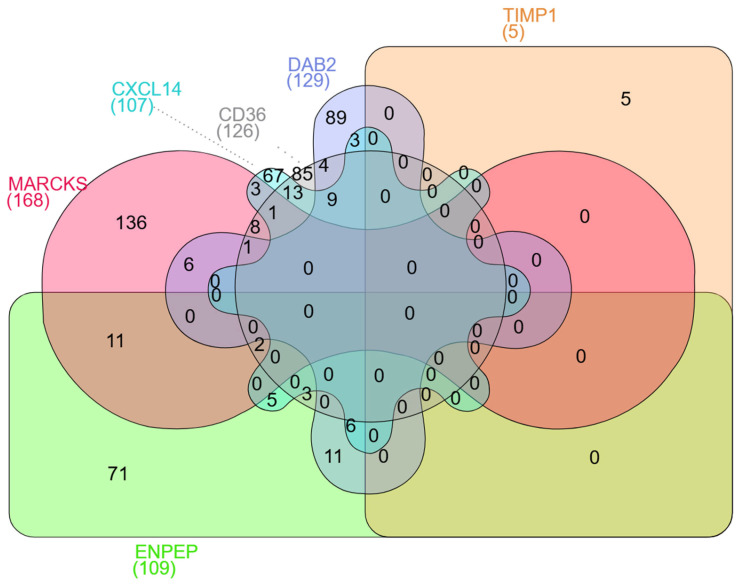
Venn diagram illustrates the overlap of predicted target genes for six EV-associated genes: **MARCKS (168 targets)**, **CXCL14 (107)**, **CD36 (126)**, **DAB2 (129)**, **ENPEP (109)**, and **TIMP1 (5)**. Numbers indicate the count of shared miRNAs among different combinations of these molecules.

The miRNAs that influence the CD36, CXCL14, DAB2, MARCKS, ENPEP, and TIMP1 gene groups are detailed in Table 2 and illustrated in the Venn diagram in Figure 13. The CD36 and CXCL14 are affected by the largest number of common miRNAs (n = 13). This is followed by the common miRNAs (n = 11 impacting the ENPEP and MARCKS as well as the ENPEP and DAB2 pairs). Among the triple gene groups, the combination of CD36, DAB2, and CXCL14 is influenced by the highest number of common miRNAs (n = 9).

### 2.10. Gene–Gene Interaction

**Figure 14:**
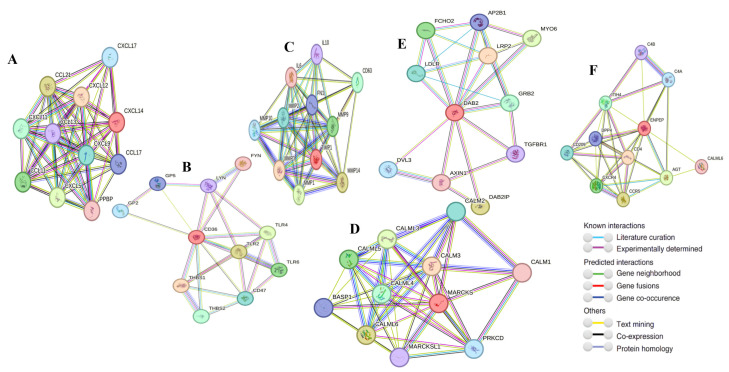
Gene–gene interaction analysis result for CXCL14 (**A**), CD36 (**B**), TIMP1 (**C**), MARCKS (**D**), DAB2 (**E**), ENPEP (**F**).

String analysis results indicate that the top ten nodes associated with CXCL14 are CXCL17, CCL21, CXCL12, CXCL11, CCL13, CXCL9, CCL17, CCL11, CXCL5, and PPBP (Figure 14A). Similarly, the top ten nodes linked to CD36 include GP2, GP5, LYN, FYN, THBS1, TRL2, TRL4, THBS2, CD47, and TRL6 (Figure 14B). For TIMP1, the top ten associated nodes are IL10, IL6, MMP10, MMP2, FN1, CD63, MMP3, MMP9, MMP1, and MMP14 (Figure 14C). The nodes most closely related to MARCKS are BASP1, CALML5, CALML3, CALML2, CALML6, CALML4, MARCKS1, PRKCD, and CALML1 (Figure 14D). In the case of DAB2, the top ten nodes are LDLR, FCHO2, AP2B1, MYO6, LRP2, GRB2, DVL3, AXIN1, DAB2IP, and TGFBR1 (Figure 14E). Lastly, the nodes associated with ENPEP are C4B, C4A, ITIH4, CD209, DPP4, CXCR4, CD4, CCR5, AGT, and CALML6 (Figure 14F).

## 3. Discussion

To the best of our knowledge, this study is among the first to integrate multiple bioinformatics platforms to investigate the expression and regulation of ENPEP, TIMP1, CD36, MARCKS, DAB2, CXCL14, and miRNAs (miR-222-3p, miR-181b-5p) in BC. Our findings underscore their potential roles as prognostic biomarkers and therapeutic targets, particularly in aggressive BC subtypes such as TNBC. These molecules were selected based on their documented relevance to BC biology and prior evidence suggesting their presence in EVs according to literature and databases such as 5.1 and ExoCarta v1.0. However, we did not analyze EV-specific datasets nor apply vesicular cargo prediction tools; thus, our findings may be interpreted as suggestive of possible EV involvement, rather than conclusive.

EVs have emerged as pivotal mediators in cancer biology, reflecting the molecular profile of their cells of origin. Specific EV-associated miRNAs, such as miR-21, miR-155, miR-222-3p, and miR-181b-5p, have been implicated in metastasis and chemoresistance in BC. These miRNAs contribute to oncogenic signaling and immune modulation and are detectable in circulation, rendering them promising candidates for liquid biopsy-based diagnosis [17,18,19]. miR-181b-5p was upregulated and showed a negative correlation with CXCL14, while miR-222-3p exhibited positive correlations with TIMP1, DAB2, and CD36. Although these associations are consistent with predicted regulatory interactions, they may be interpreted as hypothesis-generating findings that require further experimental validation.

CD36, a scavenger receptor implicated in fatty acid metabolism and angiogenesis, has been extensively investigated in the context of cancer. While it is commonly linked to metastasis and poor prognosis in various malignancies, including BC [20], our analysis revealed that CD36 was significantly downregulated in the BC cohort, particularly in ER-, PR-, HER2+, TNBC, and basal-like subtypes. Notably, CD36 expression did not exhibit a significant correlation with OS in our cohort, suggesting a potentially context-specific or subtype-limited prognostic role. The observed downregulation may be attributed to promoter hypermethylation [21], as demonstrated by our methylation analysis. It is also noteworthy that CD36 expression is often diminished in tumor-associated stroma, contributing to metabolic rewiring and tumor progression. Previous studies have indicated that reduced stromal CD36 is associated with increased tumor aggressiveness [22,23]. However, in patients with HER2+ breast cancer, elevated CD36 expression correlates with a poor prognosis [20]. Interestingly, in our BC cohort, CD36 expression levels were not associated with OS. According to an IHC investigation, the significant downregulation of CD36 in ER-, PR-, HER2+, TNBC, and basal-like breast cancer may suggest a shift in metabolic and tumor microenvironmental dynamics. These findings suggest that CD36 may function as a context-dependent modulator of fatty acid metabolism and tumor–stroma interactions in BC. Its downregulation in aggressive subtypes, particularly TNBC and basal-like tumors, supports its potential role as a subtype-specific biomarker and warrants further functional investigation.

CXCL14 expression was similarly downregulated in BC tissues, particularly in TNBC and basal-like subtypes, known for their aggressiveness and limited treatment options. Previous research indicates that stromal CXCL14 expression serves as an independent predictor of decreased BC-specific and recurrence-free survival, especially in alpha ER- tumors [24,25]. Both in vitro and in vivo studies suggest that re-expressing CXCL14 can suppress tumor growth and metastasis by enhancing immune cell recruitment and modulating the tumor microenvironment [26]. In our dataset, this downregulation may be attributed to promoter hypermethylation and post-transcriptional regulation via miR-181b-5p. Furthermore, IHC findings suggest that CXCL14 downregulation in ER-, PR-, human epidermal growth HER2+, TNBC, and basal-like subtypes may lead to immune evasion, reduced tumor immunogenicity, and increased aggressiveness. Taken together, the consistent downregulation of CXCL14 across multiple aggressive BC subtypes and its association with shorter OS indicate that it may serve as a prognostic marker and therapeutic target, particularly through its role in immune modulation and tumor microenvironment regulation.

TIMP1, a tissue inhibitor of metalloproteinases, plays a context-dependent role in BC. While it has been implicated in extracellular matrix remodeling, proliferation, and chemoresistance, its prognostic value remains debated [27,28,29,30]. In contrast to these studies, another study suggested that low TIMP1 expression levels may serve as an independent prognostic and predictive biomarker for breast cancer [31]. Our analysis showed TIMP1 upregulation in luminal and TNBC subtypes and an association with improved OS, contrasting with some studies linking high TIMP1 levels to poor outcomes. This discrepancy may reflect the dual role of TIMP1: it may act as a tumor suppressor by stabilizing the ECM in certain subtypes while promoting immune evasion or resistance in others. IHC data further revealed lower TIMP1 expression in HER2+, ER–, and PR– tumors. These findings highlight the need for subtype-stratified evaluation to accurately assess TIMP1’s biomarker potential.

DAB2, known as a tumor suppressor and adaptor protein, is involved in TGF-β signaling, cytoskeletal regulation, and immune modulation [32]. Consistent with its documented role in various cancers [32], our analysis revealed a significant downregulation of DAB2 in BC tissues, especially in TNBC. This reduction is associated with decreased OS. Promoter hypermethylation may play a crucial role in silencing DAB2 expression. Notably, DAB2 affects the endocytosis of TGF-β receptors and contributes to immune tolerance regulation through Treg cell induction [32,33]. Its loss could lead to unregulated TGF-β signaling, promoting a tumor-friendly microenvironment characterized by immune suppression and metastatic potential [34,35]. The low expression of DAB2 in HER2-, ER-, and TNBC tumors further highlights its potential role as a tumor suppressor in aggressive BC subtypes. Interestingly, our findings also showed a positive correlation between DAB2 and miR-222-3p expression, indicating complex regulatory dynamics that merit further investigation. The downregulation of DAB2, along with its known tumor-suppressive and immunomodulatory functions, suggests a mechanistic contribution to tumor progression in TNBC. Its epigenetic silencing may thus represent a key event in disrupting TGF-β signaling and immune regulation in aggressive BC phenotypes.

Although miR-222-3p is often identified as an oncomiR in various cancers [36], it was found to be downregulated in our BC cohort. This observation contradicts previous reports linking elevated miR-222-3p expression with tumor proliferation, metastasis, and drug resistance. However, low serum levels of miR-222-3p have also been associated with poor prognosis in BC [37]. Our study confirmed its upregulation in BC, particularly in TNBC, which aligns with its known roles in promoting proliferation and chemoresistance. Given its association with EVs and expression in immune cells, miR-222-3p may serve as both a biomarker and an effector in tumor-immune interactions. These findings underscore its significance in the development of targeted therapies. They suggest a complex and potentially subtype-specific association pattern for miR-222-3p in BC. Nevertheless, due to the absence of experimental confirmation, its role remains speculative and requires functional validation. Although traditionally characterized as an oncomiR, its downregulation and correlation with tumor suppressor genes in this cohort may indicate a subtype-specific functional shift. The upregulation of miR-181b-5p and its negative correlation with CXCL14 expressions suggest a potential role in the post-transcriptional suppression of tumor-suppressive pathways. These observations support its investigation as a candidate mediator of immune evasion and metastatic progression in BC.

In our study, we identified promoter hypermethylation as a significant regulatory mechanism, particularly influencing the expression of CD36, CXCL14, and DAB2. The epigenetic silence of these tumor suppressors may facilitate tumor progression, immune evasion, and metastasis. Methylation-based repression patterns can differ among tumor subtypes, suggesting their potential as biomarkers for prognosis and therapeutic stratification [38,39]. The identification of promoter hypermethylation in CD36, CXCL14, and DAB2 underscores the role of epigenetic silencing in BC pathogenesis. These findings also emphasize the potential of methylation profiling as a complementary biomarker strategy for stratifying tumor subtypes.

STRING network analysis revealed that our target genes are involved in critical cancer-related processes, including immune modulation, inflammation, cell migration, and extracellular matrix remodeling. Enrichment analyses showed that TIMP1, ENPEP, CD36, and CXCL14 contribute to sustaining proliferative signaling. Additionally, CXCL14, DAB2, ENPEP, and MARCKS were implicated in evading growth suppressors, while TIMP1, CD36, and CXCL14 were linked to sustained angiogenesis. These processes are hallmarks of cancer and highlight the significance of our findings.

Despite the increasing interest in EVs as mediators of intercellular communication and cancer progression, the field encounters several unresolved challenges. A significant limitation is the heterogeneity of EV populations, which vary considerably in size, content, and cellular origin [40]. Currently, there is no universally accepted set of molecular markers that definitively distinguish EV subtypes, complicating efforts to attribute specific biological functions to EV cargos [41]. Moreover, it is increasingly recognized that molecules from the extracellular environment may adsorb onto EV surfaces, forming a “protein corona.” This phenomenon can influence the detection, function, and downstream interpretation of EV-associated proteins. These factors pose challenges to the consistency and reproducibility of EV studies, including those involving bioinformatics analyses [42,43]. While our study focuses on molecules previously reported to be enriched in EVs, we acknowledge that some may represent dynamic or context-dependent associations rather than core vesicular components. Further experimental validation using EV isolation and proteomic/miRNA profiling will be essential to confirm the vesicular origin and functionality of the candidate biomarkers reported here. These molecules have been implicated in EV-mediated communication that enhances metastatic potential, modulates immune responses, or facilitates resistance to therapy. For instance, TIMP1 has been shown to be packaged into EVs and promote tumor invasiveness via extracellular matrix remodeling [44,45]. Similarly, CD36 and DAB2, when carried by EVs, contribute to lipid uptake and signal transduction within the tumor stroma [34,46]. The miRNAs analyzed in our study, miR-181b-5p and miR-222-3p, are also well-characterized EV components derived from cancer cells and function as post-transcriptional regulators in recipient cells [17,47]. Thus, while our results reflect differential expression patterns and regulatory associations, the implication of these molecules in EV-mediated BC pathogenesis remains speculative and warrants experimental validation through EV isolation and profiling studies.

Overall, our findings suggest that these genes and miRNAs are central to BC pathogenesis and progression, with relevance to aggressive subtypes such as TNBC. Their involvement in essential biological processes and their potential regulation via methylation and miRNAs highlight their utility as diagnostic and therapeutic targets. Future studies should incorporate functional assays, in vitro validation, and stratified patient cohorts to further elucidate the mechanistic pathways underlying these associations and assess their clinical applicability. Importantly, the inclusion of these molecules was grounded in their consistent identification as EV cargos. Their altered expression across BC subtypes, along with prognostic implications, strengthens the hypothesis that they may participate in EV-mediated transfer of oncogenic signals or immune evasion mechanisms. Future studies should investigate whether these molecules directly modulate recipient cell behavior via EV transport.

### Limitation

This study encountered several limitations. The data were sourced from specific databases, which restricted access to comprehensive data on BC patients. Consequently, it was not feasible to perform OS analysis individually for each BC subtype, resulting in incomplete data in this regard. Additionally, the disparity in the number of normal adjacent tissue samples compared to tumor tissue samples may introduce bias. Therefore, the findings derived from these databases require validation through further studies involving homogeneous patient groups. While several of the molecules investigated in this study exhibited statistically significant differential expressions between tumor and normal breast tissues, it is important to emphasize that statistical significance does not inherently imply clinical utility as a biomarker. This is particularly relevant in large data sets such as TCGA, where even small differences may yield extremely low *p*-values. However, as illustrated in Figure 1, the expression distributions of some molecules (e.g., TIMP1, miR-181b-5p, miR-222-3p) show considerable overlap between the cancer and normal groups. This overlap suggests that these markers may yield a non-negligible rate of false positives or false negatives if used diagnostically without additional filtering criteria. For a molecule to be considered a robust biomarker, beyond statistical significance, it should demonstrate a high discriminatory power as measured by sensitivity, specificity, and preferably an AUC (Area Under the Curve) greater than 0.80 in receiver operating characteristic (ROC) analysis. Additionally, effect size (e.g., fold-change or Cohen’s d) and reproducibility across independent cohorts are essential metrics. These considerations highlight the need for further validation and classification modeling, which should be addressed in future studies incorporating cross-validation and independent datasets.

## 4. Materials and Methods

This is a bioinformatic-based study. In the TCGA-BRCA BC cohort analyzed in this study, which includes RNA-sequencing and clinical data from 1085 primary breast tumor samples and 291 normal breast tissue samples, patients were further stratified by molecular subtype (luminal A, luminal B, HER2-enriched, and triple-negative) according to PAM50 annotations provided in the TCGA database.

We used the following bioinformatic databases while evaluating prognostic features of ENPEP, TIMP1, CD36, MARCKS, DAB2, CXCL14, miR181b-5p and miR-222-3p in BC. The selection of ENPEP, TIMP1, CD36, MARCKS, DAB2, CXCL14, miR-181b-5p, and miR-222-3p was based on a dual strategy combining literature-based prioritization and bioinformatics screening. Initially, we conducted a comprehensive review of EV-related studies and BC literature to identify genes and microRNAs commonly reported to be enriched in EVs and implicated in tumor progression, immune modulation, metastasis, or angiogenesis. These molecules were further screened using public datasets and EV-focused databases to confirm their detectable expression in breast cancer tissues and their availability across multiple omics platforms. Differential expression patterns and previously reported roles in EV-mediated intercellular communication (especially for miR-181b-5p and miR-222-3p) further supported their inclusion in the current study. By focusing on these specific candidates, we aim to ensure both biological relevance and analytical feasibility for downstream functional and clinical correlation analyses.

**Expression profile analysis of ENPEP, TIMP1, CD36, MARCKS, DAB2, and CXCL14**

**The access date:** 15 February 2025

The GEPIA2 (http://gepia2.cancer-pku.cn/, accessed on 15 February 2025) web database was used for expression profile analysis. GEPIA2 is an enhanced portion of the web server that offers more detailed gene expression analysis, including transcription-level quantification, analysis of specific cancer subtypes, and the ability for users to upload their own RNA-seq fragments to separate with existing datasets [48]. With the GEPIA2 web server, it is possible to compare the expression levels of genes examined in both tumoral tissue and adjacent healthy tissue. It is also possible to compare the expression levels of the gene or genes examined in cancer subtypes according to the type of cancer. In our BC cohort, we compared the expression levels of ENPEP, TIMP1, CD36, MARCKS, DAB2, and CXCL14 genes with the healthy tissue adjacent (n = 291) to the tumor tissue (n = 1085). We also used the Human Protein Atlas (HPA) (https://www.proteinatlas.org/, accessed on 15 February 2025) to confirm the expression results obtained from GEPIA2. This section of the Human Protein Atlas highlights the expression patterns of genes in human tissues, analyzing both mRNA and protein levels. Protein expression data from 44 normal human tissue types is obtained through antibody-based protein profiling, utilizing conventional and multiplex immunohistochemistry techniques [49].

**Targeted expression analysis and gene correlation targeted analysis**

**The access date:** 15 February 2025

We utilized bc-GenExMiner v5.1 web tool for analysis of targeted expression and gene correlation. bc-GenExMiner v5.1 is a statistical analysis tool designed for examining annotated breast cancer transcriptomic data, including DNA microarrays (n = 11,552) and RNA-seq datasets (n = 5023). It enables users to investigate the expression of specific genes of interest in the context of breast cancer [50,51].

**The methylation status analysis of ENPEP, TIMP1, CD36, MARCKS, DAB2, and CXCL14**

**The access date:** 15 February 2025

UALCAN (https://ualcan.path.uab.edu/, accessed on 15 February 2025) is a comprehensive, user-friendly, interactive web resource for analyzing cancer transcriptomic data. It aims to facilitate the exploration of TCGA (The Cancer Genome Atlas) data and enable researchers to analyze gene expression profiles and perform in-depth analyses of various cancer types. UALCAN provides insight into the methylation status of gene promoters. Users can compare methylation levels between tumor and normal samples. It allows us to study the correlation between expression levels of two genes. The server is useful for identifying potential gene interactions and pathways [52]. We performed promoter region methylation analyses (tumor tissue n = 793), (normal tissue n = 97) with the UALCAN web server and examined the expression profiles of other input genes in BC, including ENPEP, TIMP1, CD36, MARCKS, DAB2, and CXCL14. Additionally, we also compared the BC subtypes (luminal (n = 566), HER2+ (n = 37), triple negative breast cancer (TNBC) (n = 116)) expression profiles, including ENPEP, TIMP1, CD36, MARCKS, DAB2, and CXCL14, with each other and normal adjacent tissue (n = 114).

**The survival analysis of ENPEP, TIMP1, CD36, MARCKS, DAB2, and CXCL14**

**The access date:** 15 February 2025

KM Plotter (Kaplan–Meier Plotter) (https://kmplot.com/analysis/index.php?p=home, accessed on 15 February 2025) is an online tool designed to assess the effect of gene survival on various cancers using clinical data. It enables researchers to conduct meta-analyses of gene expression data to identify potential prognostic biomarkers. Users can generate Kaplan–Meier survival plots to visualize the relationship between gene expression levels and patient survival outcomes. It integrates data from several high-quality sources such as GEO (Gene Expression Omnibus), EGA (European Genome-phenome Archive), and TCGA and combines clinical data with gene expression profiles to provide a comprehensive analysis [53].

**The relationship between MicroRNA vs. BC and MicroRNA vs. RNA**

**The access date:** 15 February 2025

The ENCORI Pan-Cancer Analysis Platform (https://rnasysu.com/encori/index.php, accessed on 15 February 2025) is developed to decode Pan-Cancer Networks involving lncRNAs, miRNAs, pseudogenes, snoRNAs, RNA-binding proteins (RBPs), and all protein-coding genes. It achieves this by analyzing their expression profiles across 32 cancer types, utilizing data from approximately 10,000 RNA-seq and 9900 miRNA-seq samples integrated from the TCGA project [54].

**Enrichment analysis of ENPEP, TIMP1, CD36, MARCKS, DAB2, and CXCL14**

**The access date:** 15 February 2025

Differentially expressed gene (DEG) analysis was conducted using the Enrichr-KG web tool (https://maayanlab.cloud/enrichr-kg, accessed on 15 February 2025) across several categories: Gene Ontology (GO) biological process, Kyoto Encyclopedia of Genes and Genomes (KEGG), Jensen_DISEASES for exploring disease-gene associations, and DisGeNET for compiling data on genes and variants linked to diseases. The analysis was limited to the top 20 terms, with statistical significance defined as *p* < 0.05 [55].

**Cancer Hallmark Enrichment plot and**

**The access date:** 15 February 2025

The Cancer Hallmark Enrichment tool (https://cancerhallmarks.com/, accessed on 15 February 2025) compiles a consensus list of cancer hallmark genes by integrating 6763 genes from various mapping resources. CancerHallmarks.com leverages the hallmark concept as a powerful organizational framework, linking genes to their corresponding biological functions [56].

**Tumor, Normal, and Metastatic (TNM) Plot Analysis**

**The access date:** 15 February 2025

The Tumor, Normal, and Metastatic (TNM) plot database provides users with a real-time comparison of gene expression changes across tumor, normal, and metastatic tissues for all genes, utilizing multiple platforms. The multiple gene analysis page provides an overview of the selected gene set in the selected tissue using gene chip-based data. Accessible without registration at www.tnmplot.com (accessed on 15 February 2025) the portal features three distinct analysis options. Among these, the pan-cancer analysis tool allows for a simultaneous comparison of normal and tumor samples across 22 different tissue types [57].

**Prediction of miRNAs associated with genes**

**The access date:** 15 February 2025

We used the MicroRNA Target Prediction Database (miRDB) (https://mirdb.org/, accessed on 15 February 2025) [58] and TargetScan 8.0 (https://www.targetscan.org/vert_80/, accessed on 15 February 2025) to identify and predict the target genes of differentially expressed miRNAs [58,59].

**Gene–gene interaction analysis**

**The access date:** 15 February 2025

The STRING database (https://string-db.org/, accessed on 15 February 2025) was employed to elucidate the interactions and potential mechanisms involving ENPEP, TIMP1, CD36, MARCKS, DAB2, and CXCL14 along with other related proteins. STRING systematically predicts protein–protein interactions by considering both functional and physical associations. The data were sourced from various origins, including automated text mining of the scientific literature, computational predictions based on co-expression patterns and conserved genomic contexts, as well as curated interaction experiment databases. These interactions undergo rigorous evaluation, scoring, and hierarchical orthology-based transfer to organisms with limited prior research [60].

## 5. Conclusions

This integrative bioinformatics study underscores the pivotal roles of EV-associated genes and miRNAs, specifically ENPEP, TIMP1, CD36, MARCKS, DAB2, CXCL14, miR-222-3p, and miR-181b-5p, in the pathogenesis and progression of BC. Our findings indicate that these molecules are not only differentially expressed across molecular subtypes of BC, particularly in aggressive forms such as TNBC, but also engage in key oncogenic processes, including immune modulation, extracellular matrix remodeling, angiogenesis, and metastasis.

We observed significant expression changes in CD36, CXCL14, DAB2, and TIMP1, often associated with promoter methylation or regulatory miRNAs. Notably, the expression levels of TIMP1, DAB2, and CXCL14 were positively correlated with patient OS, whereas miR-181b-5p may serve as a negative prognostic factor by downregulating tumor suppressor genes. The downregulation of CD36 and CXCL14, alongside the upregulation of TIMP1, underscores the complex and context-dependent roles these genes play in BC biology.

Our enrichment and network analyses further corroborate that these genes and miRNAs—selected due to their reported association with extracellular vesicles—are functionally implicated in core hallmarks of cancer, providing a compelling rationale for their consideration as potential biomarkers or therapeutic targets. This is particularly crucial in TNBC, where effective targets remain scarce. Their vesicular context suggests that they may exert systemic effects by modulating recipient cells through EV-mediated pathways, highlighting their translational potential in diagnosis and therapy.

Collectively, these findings lay the groundwork for future functional studies and clinical validation efforts to explore the diagnostic, prognostic, and therapeutic potential of these EV-related molecules in BC. A deeper understanding of their regulatory mechanisms, especially those involving promoter methylation and miRNA interaction, could ultimately contribute to more personalized and effective treatment strategies.

## Figures and Tables

**Table 1:** Gene set enrichment analysis statistical results for cancer hallmarks.

Cancer Hallmark	Overlap	*p*-Value	Adjusted *p*-Value	Odds Ratio	Hallmark vs. Hallmark	Genes
Sustaining Proliferative Signaling	4/3574	0.03172	0.06175	5.76	0.69	TIMP1; ENPEP; CD36; CXCL14
Genome Instability	0/1	nan	1.0	nan	0.0	nan
Evading Growth Suppressors	5/3288	0.00248	0.01012	13.08	0.93	TIMP1; CXCL14; DAB2; ENPEP; MARCKS
Evading Immune Destruction	1/749	0.26464	0.26464	5.19	0.82	CXCL14
Sustained Angiogenesis	3/796	0.00264	0.01012	17.9	2.31	TIMP1; CD36; CXCL14
Tissue Invasion and Metastasis	3/2318	0.05115	0.07672	5.48	0.79	TIMP1; ENPEP; CD36
Tumor-Promoting Inflammation	2/769	0.03431	0.06175	10.3	1.6	CD36; CXCL14
Resisting Cell Death	4/1941	0.00337	0.01012	12.13	1.26	TIMP1; DAB2; CD36; CXCL14
Reprogramming Energy Metabolism	1/740	0.26185	0.26464	5.26	0.83	CD36
Replicative Immortality	1/547	0.19983	0.25692	7.21	1.12	CXCL14

**Table 2:** The miRNAs related to gene groups.

Gene Groups	miRNAs Related to Genes
CD36 and DAB2	hsa-miR-4729, hsa-miR-651-5p, hsa-miR-5579-3p, hsa-miR-4520-2-3p
DAB2 and CXCL14	hsa-miR-4713-5p, hsa-miR-548t-5p, hsa-miR-548az-5p
CD36 and CXCL14	hsa-miR-4719, hsa-miR-340-5p, hsa-miR-651-3p, hsa-miR-4477b, hsa-miR-1277-5p, hsa-miR-150-5p, hsa-miR-153-5p, hsa-miR-6854-5p, hsa-miR-548ar-3p, hsa-miR-548a-3p, hsa-miR-548f-3p, hsa-miR-548az-3p, hsa-miR-548e-3p
MARCKS and CXCL14	hsa-miR-3121-5p, hsa-miR-5088-3p, hsa-miR-3143
ENPEP and CXCL14	hsa-miR-3163, hsa-miR-3662, hsa-miR-106a-3p, hsa-miR-142-5p, hsa-miR-5590-3p
ENPEP and CD36	hsa-miR-548e-5p, hsa-miR-4659a-3p, hsa-miR-4659b-3p
CD36 and MARCKS	hsa-miR-1537-5p, hsa-miR-4276, hsa-miR-545-3p, hsa-miR-134-3p, hsa-miR-3124-3p, hsa-miR-655-3p, hsa-miR-374c-5p, hsa-miR-576-5p
ENPEP and MARCKS	hsa-miR-9-5p, hsa-miR-641, hsa-miR-3617-5p, hsa-miR-520d-5p, hsa-miR-524-5p, hsa-miR-551b-5p, hsa-miR-4668-3p, hsa-miR-3606-3p, hsa-miR-513a-3p, hsa-miR-513c-3p, hsa-miR-4490
MARCKS and DAB2	hsa-miR-6878-5p, hsa-miR-6825-5p, hsa-miR-664b-3p, hsa-miR-579-3p, hsa-miR-3925-3p, hsa-let-7c-3p
ENPEP and DAB2	hsa-miR-1297, hsa-miR-26a-5p, hsa-miR-26b-5p, hsa-miR-4465, hsa-miR-376a-5p, hsa-miR-3150b-3p, hsa-miR-4784, hsa-miR-3977, hsa-miR-6768-3p, hsa-miR-15b-3p, hsa-miR-4428
ENPEP and DAB2 and CXCL14	hsa-miR-186-5p, hsa-miR-320d, hsa-miR-320c, hsa-miR-320b, hsa-miR-4429, hsa-miR-9-3p
CD36 and MARCKS and DAB2	hsa-miR-5696
CD36, MARCKS and CXCL14	hsa-miR-410-3p
CD36, DAB2 and CXCL14	hsa-miR-539-5p, hsa-miR-526b-3p, hsa-miR-93-5p, hsa-miR-20b-5p, hsa-miR-106b-5p, hsa-miR-20a-5p, hsa-miR-17-5p, hsa-miR-519d-3p, hsa-miR-106a-5p
ENPEP, CD36 and MARCKS	hsa-miR-4328, hsa-miR-548c-3p

## Data Availability

The data used in this study were obtained from the public database TCGA and others.

## Abbreviations

The following abbreviations are used in this manuscript:

Abbreviation	Full Name
BC	Breast cancer
EV	Extracellular vesicle
miRNA	MicroRNA
lncRNA	Long non-coding RNA
IHC	Immunohistochemistry
OS	Overall survival
GEPIA2	Gene Expression Profiling Interactive Analysis 2
HPA	Human Protein Atlas
UALCAN	University of Alabama at Birmingham CANcer data analysis portal
TNM	Tumor, Normal, and Metastatic
KM Plotter	Kaplan–Meier Plotter
ENCORI	Encyclopedia of RNA Interactomes
KEGG	Kyoto Encyclopedia of Genes and Genomes
GO	Gene Ontology
STRING	Search Tool for the Retrieval of Interacting Genes/Proteins
PAM50	Prediction Analysis of Microarray 50
TNBC	Triple negative breast cancer
ER	Estrogen receptor
PR	Progesterone receptor
HER2	Human Epidermal Growth Factor Receptor 2
TIMP1	Tissue inhibitor of metalloproteinases 1
CD36	Cluster of Differentiation 36
MARCKS	Myristoylated Alanine-Rich C Kinase Substrate
DAB2	Disabled Homolog 2
CXCL14	C-X-C Motif Chemokine Ligand 14
ENPEP	Glutamyl Aminopeptidase
miR-181b-5p	MicroRNA-181b-5p
miR-222-3p	MicroRNA-222-3p
miRDB	MicroRNA Target Prediction Database
TargetScan	TargetScan microRNA target prediction tool
TCGA	The Cancer Genome Atlas

## References

[1] Lopez-Gonzalez L., Sanchez C.A., Sanchez C.C., Roberts C.E., Espinosa J., Pekarek T., Fraile-Martinez O., García-Montero C., Rodriguez-Slocker A.M., Jiménez-Álvarez L. (2024). Exploring Biomarkers in Breast Cancer: Hallmarks of Diagnosis, Treatment, and Follow-Up in Clinical Practice. Medicina.

[2] Wilkinson L., Gathani T. (2022). Understanding breast cancer as a global health concern. Br. J. Radiol..

[3] Panigrahi L., Samal P., Sahoo S., Sahoo B., Kumar P.A., Mahanta S., Rath S.K., Arakha M. (2024). Nanoparticle-mediated diagnosis, treatment, and prevention of breast cancer. Nanoscale Adv..

[4] Zhang X., Wang C., Yu J., Bu J., Ai F., Wang Y., Lin J., Zhu X. (2023). Extracellular vesicles in the treatment and diagnosis of breast cancer: A status update. Front. Endocrinol..

[5] Bandu R., Oh J., Kim K. (2024). Extracellular vesicle proteins as breast cancer biomarkers: Mass spectrometry-based analysis. Proteomics.

[6] Lopez K., Lai S., Lopez G.E., Dávila R., Shuck S. (2023). Extracellular vesicles: A dive into their role in the tumor microenvironment and cancer progression. Front. Cell Dev. Biol..

[7] Andre M., Caobi A., Miles J., Vashist A., Ruiz M., Raymond A. (2024). Diagnostic potential of exosomal extracellular vesicles in oncology. BMC Cancer.

[8] Kumar M., Baba S., Sadida H., Marzooqi S., Jerobin J., Altemani H., Algehainy N., Alanazi M.A., Abou-Samra A.B., Kumar R. (2024). Extracellular vesicles as tools and targets in therapy for diseases. Signal Transduct. Target. Ther..

[9] Barile L., Vassalli G. (2017). Exosomes: Therapy delivery tools and biomarkers of diseases. Pharmacol Ther..

[10] Doyle L.M., Wang M.Z. (2019). Overview of Extracellular Vesicles, Their Origin, Composition, Purpose, and Methods for Exosome Isolation and Analysis. Cells.

[11] van Niel G., D’Angelo G., Raposo G. (2018). Shedding light on the cell biology of extracellular vesicles. Nat. Rev. Mol. Cell Biol..

[12] Xu R., Rai A., Chen M., Suwakulsiri W., Greening D.W., Simpson R.J. (2018). Extracellular vesicles in cancer-implications for future improvements in cancer care. Nat. Rev. Clin. Oncol..

[13] Kalra H., Simpson R.J., Ji H., Aikawa E., Altevogt P., Askenase P., Bond V.C., Borràs F.E., Breakefield X., Budnik V. (2012). Vesiclepedia: A compendium for extracellular vesicles with continuous community annotation. PLoS Biol..

[14] Mathivanan S., Fahner C.J., Reid G.E., Simpson R.J. (2012). ExoCarta 2012: Database of exosomal proteins, RNA and lipids. Nucleic Acids Res..

[15] Kalluri R., LeBleu V.S. (2020). The biology, function, and biomedical applications of exosomes. Science.

[16] Hutter C., Zenklusen J.C. (2018). The Cancer Genome Atlas: Creating Lasting Value beyond Its Data. Cell.

[17] Ahmed S., Espinoza-Sánchez N., El-Damen A., Fahim S., Badawy M., Greve B., El-Shinawi M., Götte M., Ibrahim S.A. (2021). Small extracellular vesicle-encapsulated miR-181b-5p, miR-222-3p and let-7a-5p: Next generation plasma biopsy-based diagnostic biomarkers for inflammatory breast cancer. PLoS ONE.

[18] Warren L., Guo H., Regan M., Nakhlis F., Yeh E., Jacene H., Hirshfield-Bartek J., Overmoyer B.A., Bellon J.R. (2015). Inflammatory breast cancer and development of brain metastases: Risk factors and outcomes. Breast Cancer Res. Treat..

[19] Uemura M., French J., Hess K., Liu D., Raghav K., Hortobagyi G., Arun B.K., Valero V., Ueno N.T., Alvarez R.H. (2018). Development of CNS metastases and survival in patients with inflammatory breast cancer. Cancer.

[20] Guerrero-Rodríguez S., Mata-Cruz C., Pérez-Tapia S., Velasco-Velázquez M. (2022). Role of CD36 in cancer progression, stemness, and targeting. Front. Cell Dev. Biol..

[21] Stirzaker C., Song J., Davidson B., Clark S. (2004). Transcriptional gene silencing promotes DNA hypermethylation through a sequential change in chromatin modifications in cancer cells. Cancer Res..

[22] DeFilippis R., Chang H., Dumont N., Rabban J., Chen Y., Fontenay G., Berman H.K., Gauthier M.L., Zhao J., Hu D. (2012). CD36 repression activates a multicellular stromal program shared by high mammographic density and tumor tissues. Cancer Discov..

[23] Nath A., Chan C. (2016). Genetic alterations in fatty acid transport and metabolism genes are associated with metastatic progression and poor prognosis of human cancers. Sci. Rep..

[24] Sjöberg E., Augsten M., Bergh J., Jirström K., Östman A. (2016). Expression of the chemokine CXCL14 in the tumour stroma is an independent marker of survival in breast cancer. Br. J. Cancer.

[25] Gibbs C., So J., Ahad A., Michalowski A., Son D., Li Y. (2022). CXCL14 Attenuates Triple-Negative Breast Cancer Progression by Regulating Immune Profiles of the Tumor Microenvironment in a T Cell-Dependent Manner. Int. J. Mol. Sci..

[26] Gu X., Ou Z., Lin F., Yang X., Luo J., Shen Z., Shao Z. (2012). Expression of CXCL14 and its anticancer role in breast cancer. Breast Cancer Res. Treat..

[27] Schrohl A., Meijer-van G.M., Holten-Andersen M., Christensen I., Look M., Mouridsen H., Brünner N., Foekens J.A. (2006). Primary tumor levels of tissue inhibitor of metalloproteinases-1 are predictive of resistance to chemotherapy in patients with metastatic breast cancer. Clin. Cancer Res. Off. J. Am. Assoc. Cancer Res..

[28] Agnello L., d’Argenio A., Caliendo A., Nilo R., Zannetti A., Fedele M., Camorani S., Cerchia L. (2023). Tissue Inhibitor of Metalloproteinases-1 Overexpression Mediates Chemoresistance in Triple-Negative Breast Cancer Cells. Cells.

[29] Nabholz J., Abrial C., Mouret-Reynier M., Dauplat M., Weber B., Gligorov J., Forest A.M., Tredan O., Vanlemmens L., Petit T. (2014). Multicentric neoadjuvant phase II study of panitumumab combined with an anthracycline/taxane-based chemotherapy in operable triple-negative breast cancer: Identification of biologically defined signatures predicting treatment impact. Ann. Oncol..

[30] Cheng G., Fan X., Hao M., Wang J., Zhou X., Sun X. (2016). Higher levels of TIMP-1 expression are associated with a poor prognosis in triple-negative breast cancer. Mol. Cancer.

[31] Keskin S.E., Akkoyunlu D.S., Yılmaz M., Şimşek T., Güler S., Çine N., Cantürk Z., Savlı H. (2023). Quantification of MMP-2 and TIMP-1 expressions in breast cancer. İstanb. J. Pharm..

[32] Xu S., Zhu J., Wu Z. (2014). Loss of Dab2 Expression in Breast Cancer Cells Impairs Their Ability to Deplete TGF-β and Induce Tregs Development via TGF-β. PLoS ONE.

[33] Massagué J. (2008). TGFbeta in Cancer. Cell.

[34] Price Z., Lokman N., Yoshihara M., Kajiyama H., Oehler M., Ricciardelli C. (2023). Disabled-2 (DAB2): A Key Regulator of Anti- and Pro-Tumorigenic Pathways. Int. J. Mol. Sci..

[35] Papageorgis P., Stylianopoulos T. (2015). Role of TGFβ in regulation of the tumor microenvironment and drug delivery (Review). Int. J. Oncol..

[36] Wang D., Sang Y., Sun T., Kong P., Zhang L., Dai Y., Cao Y., Tao Z., Liu W. (2021). Emerging roles and mechanisms of microRNA-222-3p in human cancer (Review). Int. J. Oncol..

[37] Wang Y., Yin W., Lin Y., Yin K., Zhou L., Du Y., Yan T., Lu J. (2018). Downregulated circulating microRNAs after surgery: Potential noninvasive biomarkers for diagnosis and prognosis of early breast cancer. Cell Death Discov..

[38] Costello J., Frühwald M., Smiraglia D., Rush L., Robertson G., Gao X., Wright F.A., Feramisco J.D., Peltomäki P., Lang J.C. (2020). Aberrant CpG-island methylation has non-random and tumour-type-specific patterns. Nat. Genet..

[39] Esteller M., Corn P., Baylin S., Herman J. (2001). A gene hypermethylation profile of human cancer. Cancer Res..

[40] Willms E., Cabañas C., Mäger I., Wood M.J.A., Vader P. (2018). Extracellular Vesicle Heterogeneity: Subpopulations, Isolation Techniques, and Diverse Functions in Cancer Progression. Front. Immunol..

[41] Kowal J., Arras G., Colombo M., Jouve M., Morath J.P., Primdal-Bengtson B., Dingli F., Loew D., Tkach M., Théry C. (2016). Proteomic comparison defines novel markers to characterize heterogeneous populations of extracellular vesicle subtypes. Proc. Natl. Acad. Sci. USA.

[42] Tóth E.Á., Turiák L., Visnovitz T., Cserép C., Mázló A., Sódar B.W., Försönits A.I., Petővári G., Sebestyén A., Komlósi Z. (2021). Formation of a protein corona on the surface of extracellular vesicles in blood plasma. J. Extracell. Vesicles.

[43] Hallal S., Tűzesi Á., Grau G.E., Buckland M.E., Alexander K.L. (2022). Understanding the extracellular vesicle surface for clinical molecular biology. J. Extracell. Vesicles.

[44] Jung T., Castellana D., Klingbeil P., Hernández I.C., Vitacolonna M., Orlicky D.J., Roffler S.R., Brodt P., Zöller M. (2009). CD44v6 dependence of premetastatic niche preparation by exosomes. Neoplasia.

[45] Jordan K.R., Hall J.K., Schedin T., Borakove M., Xian J.J., Dzieciatkowska M., Lyons T.R., Schedin P., Hansen K.C., Borges V.F. (2020). Extracellular vesicles from young women’s breast cancer patients drive increased invasion of non-malignant cells via the Focal Adhesion Kinase pathway: A proteomic approach. Breast Cancer Res..

[46] Zhou X., Su M., Lu J., Li D., Niu X., Wang Y. (2024). CD36: The Bridge between Lipids and Tumors. Molecules.

[47] enito-Martín A., Jasiulionis M.G., García-Silva S. (2023). Extracellular vesicles and melanoma: New perspectives on tumor microenvironment and metastasis. Front. Cell Dev. Biol..

[48] Tang Z., Kang B., Li C., Chen T., Zhang Z. (2019). GEPIA2: An enhanced web server for large-scale expression profiling and interactive analysis. Nucleic Acids Res..

[49] Uhlen M., Zhang C., Lee S., Sjöstedt E., Fagerberg L., Bidkhori G., Benfeitas R., Arif M., Liu Z., Edfors F. (2017). A pathology atlas of the human cancer transcriptome. Science.

[50] Jézéquel P., Frénel J., Campion L., Guérin-Charbonnel C., Gouraud W., Ricolleau G., Campone M. (2013). bc-GenExMiner 3.0: New mining module computes breast cancer gene expression correlation analyses. Database.

[51] Jézéquel P., Gouraud W., Ben A.F., Guérin-Charbonnel C., Juin P., Lasla H., Campone M. (2021). bc-GenExMiner 4.5: New mining module computes breast cancer differential gene expression analyses. Database.

[52] Chandrashekar D., Bashel B., Balasubramanya S., Creighton C., Ponce-Rodriguez I., Chakravarthi B., Varambally S. (2017). UALCAN: A Portal for Facilitating Tumor Subgroup Gene Expression and Survival Analyses. Neoplasia.

[53] Nagy Á., Lánczky A., Menyhárt O., Győrffy B. (2018). Validation of miRNA prognostic power in hepatocellular carcinoma using expression data of independent datasets. Sci. Rep..

[54] Li J., Liu S., Zhou H., Qu L., Yang J. (2014). starBase v2.0: Decoding miRNA-ceRNA, miRNA-ncRNA and protein–RNA interaction networks from large-scale CLIP-Seq data. Nucleic Acids Res..

[55] Evangelista J., Xie Z., Marino G., Nguyen N., Clarke D., Ma’ayan A. (2023). Enrichr-KG: Bridging enrichment analysis across multiple libraries. Nucleic Acids Res..

[56] Menyhart O., Kothalawala W., Győrffy B. (2024). A gene set enrichment analysis for the cancer hallmarks. J. Pharm. Anal..

[57] Bartha Á., Győrffy B. (2021). TNMplot.com: A Web Tool for the Comparison of Gene Expression in Normal, Tumor and Metastatic Tissues. Int. J. Mol. Sci..

[58] Chen Y., Wang X. (2020). miRDB: An online database for prediction of functional microRNA targets. Nucleic Acids Res..

[59] McGeary S.E., Lin K.S., Shi C.Y., Pham T.M., Bisaria N., Kelley G.M., Bartel D.P. (2019). The biochemical basis of microRNA targeting efficacy. Science.

[60] Szklarczyk D., Kirsch R., Koutrouli M., Nastou K., Mehryary F., Hachilif R., Gable A.L., Fang T., Doncheva N.T., Pyysalo S. (2023). The STRING database in 2023: Protein-protein association networks and functional enrichment analyses for any sequenced genome of interest. Nucleic Acids Res..

